# Bat population recoveries give insight into clustering strategies during hibernation

**DOI:** 10.1186/s12983-020-00370-0

**Published:** 2020-09-01

**Authors:** Natália Martínková, Stuart J. E. Baird, Vlastislav Káňa, Jan Zima

**Affiliations:** 1 Institute of Vertebrate Biology, Czech Academy of Sciences, Květná 8, Brno, 60365 Czechia; 2 RECETOX, Masaryk University, Kamenice 753/5, Brno, 62500 Czechia; 3 Museum Blanenska, Zámek 1, Blansko, 67801 Czechia

**Keywords:** Chiroptera, Hibernation, Population size, Clustering behaviour, Winter activity

## Abstract

**Background:**

Behaviour during hibernation contributes to energy conservation in winter. Hibernating bats select roosts with respect to physiological and environmental stressors, available local microclimate and species-specific requirements.

**Results:**

We found that, in the period between 1977 and 2018, hibernating *Myotis myotis* and *Rhinolophus hipposideros* bats showed exponential population growth. The growth rates, corrected for local winter seasonal severity and winter duration, were equal to 10 and 13%, respectively. While *R. hipposideros* only utilised the thermally stable and, at survey time, warmer corridors in the hibernaculum, an increasing proportion of *M. myotis* roosted in the thermally stable corridors as their abundance increased. About 14% of all hibernating *M. myotis* displayed solitary roosting, irrespective of other covariates. Those bats that clustered together formed progressively larger clusters with increasing abundance, particularly in cold corridors. We found no statistically significant relationship for clustering behaviour or cluster size with winter severity or winter duration.

**Conclusions:**

Abundance of hibernating bats is increasing in Central Europe. As the number of *M. myotis* bats increases, thermally unstable corridors become saturated with large clusters and the animals begin to roost deeper underground.

## Background

Bat species in the temperate zone hibernate to save energy during the winter, when food is unavailable and environmental conditions are unfavourable. Hibernating animals in torpor reduce their metabolism, lower their body temperature close to the ambient temperature, slow their heart rate and breathing frequency, and modulate the immune response [1, 2]. To reduce exposure to outside environmental conditions and predation risk, animals use natural caves, artificial galleries, cellars and other suitable places in buildings, rock crevices or hollow trees as shelters.

The phenology of hibernation is a species-specific endogenous process linked to the photoperiod, sex, age and local weather with great phenotypic plasticity [3, 4]. As a result, individuals enter hibernacula over a period of several weeks, when their numbers on site fluctuate markedly and individual torpor bouts are short [5–7]. Towards the end of hibernation, a similar activity occurs, bats change roosts in hibernacula more frequently, move closer to the entrance of underground spaces, and might emerge on warm nights [8, 9]. The direction of draught at the hibernaculum entrance can signal current weather conditions and thus affect torpor bouts or emergence from hibernation in spring [10].

The choice of a specific roost in a hibernaculum is important for successful hibernation. For each bat, laboratory experiments show that there exists a minimal torpor temperature, usually between 1 and 10 ^∘^C, where its energy expenditure is lowest [2]. Hibernating at lower than critical temperatures requires thermoregulation to prevent freezing and at higher temperatures bats conserve less energy resources [11, 12]. Microclimate at the specific roost thus affects long-term energy expenditure during torpor. Bats hibernating in tree hollows, under bark, or in shallow rock crevices face high risk that the ambient temperature at the hibernaculum will fluctuate daily over about 10 ^∘^C and could drop below zero [11, 13]. While deep underground hibernacula present more stable microclimates, cave-dwelling bats are able to choose different roosts, resulting in exposure to temperatures differing by more than 7 ^∘^C [14]. Movement within underground hibernacula enables the bats to change their roost microclimate.

However, arousal from torpor and flight are the most expensive processes during hibernation that cost the majority of available fat resources [15]. To alleviate the costs of arousal, some bats use social thermoregulation and prevent evaporative water loss by hibernating in closely huddled clusters [16, 17]. The size of the clusters changes with the season, with the largest numbers of individuals huddled together at the end of deep hibernation [18, 19]. Small clusters or solitary hibernation might be adaptive in presence of pathogens [20, 21].

Being immobile in torpor, cave-dwelling bats can be surveyed in hibernacula and regular monitoring of the abundance of hibernating bats has become a suitable tool for the assessment of changes in population size and for informing conservation practices [22, 23]. In addition to changes in population size, such surveys are frequently used to study chiropteran ecology, including species diversity and dominance or geographical distribution [24–36].

Survey timing consistency, global environmental changes and inherent changes to populations all influence longitudinal data in population size surveys. Timing the surveys to the periods from January to February in the Northern Hemisphere ensures that the bats have entered deep hibernation and their surveyed numbers are seasonally most stable and thus accurate. Throughout winter, bats naturally rouse from torpor every two to six weeks, and weather can induce increased activity in and out of hibernacula [8, 9, 15]. Periodic arousals from torpor, when bats excrete metabolic waste, drink, eat, mount an immune response or change roosts are least frequent in deep hibernation, making deep hibernation and days with coldest weather the ideal periods for surveys of cave-dwelling bats.

In the Czech Republic, a large database has been accumulated showing abundance dynamics of hibernating bats over long periods [37–41]. Caves in the most developed karstic area of the country, the Moravian Karst, have been the subject of intensive investigation in this respect [7, 39, 42], with the Bull Rock Cave identified as among the most important hibernating sites for bats in the region [43–45]. Abundance of bats surveyed in major hibernacula in this region correlates well with observations of bat abundance in other regions, making the most populated hibernacula excellent proxies for monitoring bat population sizes.

Each annual survey represents a snap-shot of population size that is largely influenced by stochasticity in natality, mortality and migration, as well as environmental conditions on the date of the survey. We report the trend in longitudinal data on bat survey counts and cluster size. Since bats utilise hibernacula seasonally and might be active outside on warm nights [5, 8, 9, 15], we expect the drivers affecting stochasticity in observed changes in the number of bats in a hibernaculum to include local weather conditions and timing of the survey with respect to the beginning of winter in a given year. We hypothesise that greater variation in survey counts will be observed later in the winter due to increased flight activity closer to emergence from hibernation, but decrease in bat flight activity in severe winters will result in smaller variation in survey counts. Bat species that huddle will hibernate in warm corridors and form larger clusters in severe winters to conserve energy through social thermoregulation.

## Results

Sixteen bat species were encountered in the Bull Rock Cave during the 41 years it has been surveyed (1977-2018), i.e., the lesser horseshoe bat (*Rhinolophus hipposideros*), the greater horseshoe bat (*Rhinolophus ferrumequinum*), serotine bat (*Eptesicus serotinus*), barbastelle bat (*Barbastella barbastellus*), the brown long-eared bat (*Plecotus auritus*), the grey long-eared bat (*Plecotus austriacus*), noctule bat (*Nyctalus noctula*), the common pipistrelle (*Pipistrellus pipistrellus*), the greater mouse-eared bat (*Myotis myotis*), the whiskered bat (*Myotis mystacinus*), Brandt’s bat (*Myotis brandtii*), Geoffroy’s bat (*Myotis emarginatus*), Natterer’s bat (*Myotis nattereri*), Bechstein’s bat (*Myotis bechsteinii*), the pond bat (*Myotis dasycneme*) and Daubenton’s bat (*Myotis daubentonii*) (Additional file 1). *Myotis myotis* (∼25 g, hibernating in small clusters) and *R. hipposideros* (∼7 g, hibernating solitary) were recorded every year; hence, further analysis focussed on these two species in order to fully exploit the 41 years of data available in the current study.

### Abundance changes

**Fig. 1:**
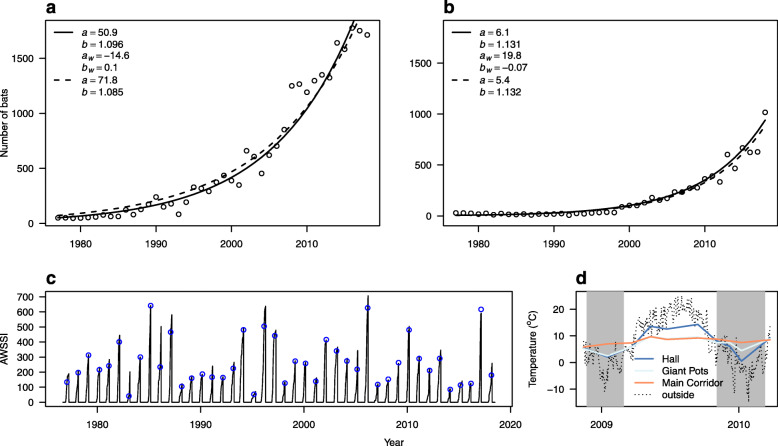
Number of hibernating bats in the Bull Rock Cave, Czech Republic, from 1977 to 2018. **a***Myotis myotis*. **b***Rhinolophus hipposideros*. Dashed line = least-squares estimate of the exponential growth model (Eq. 1), solid line = maximum likelihood estimate of the exponential growth model corrected for winter severity and winter duration up to the survey. *a* = initial population size, *b* = exponential growth parameter, *a*_*w*_ and *b*_*w*_ = intercept and slope for the influence of winter severity on growth model residuals. **c** Regional winter severity and survey dates over the course of the study. Solid line = accumulated winter season severity index (AWSSI) for the region, points = survey dates. **d** Seasonal air temperature inside the Bull Rock Cave from November 2008 to March 2010 (coloured lines, data from [46]) and daily average temperature outside reported by the Tuřany meteorological station (dotted line). Grey rectangles show winter seasons as estimated from the AWSSI calculation

**Fig. 2:**
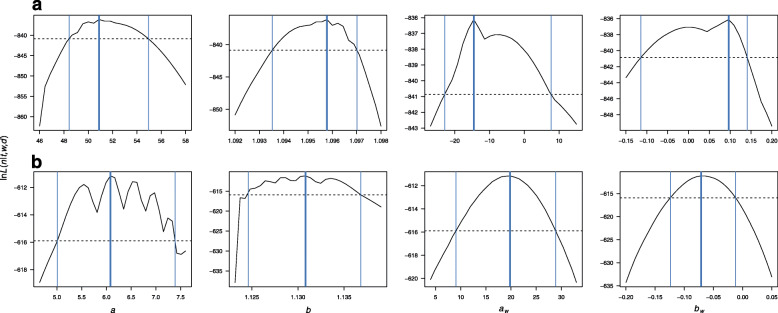
Likelihood profiles for exponential growth model parameters, corrected for winter severity and winter duration. **a***Myotis myotis*. **b***Rhinolophus hipposideros*. Thick blue line = parameter maximum likelihood estimate, thin blue lines = 95% confidence intervals of parameter estimates, dashed line = log-likelihood delimiting 95% confidence intervals of parameter estimates, *a* = initial population size, *b* = exponential growth parameter, *a*_*w*_ and *b*_*w*_ = intercept and slope of influence of winter severity on growth model residuals

The available data indicate that hibernating bat abundance has increased steadily over the survey period (Fig. 1, Additional file 1). In general, surveying commenced when winter severity was approaching its annual maximum (Fig. 1c), with winter duration up to the survey date being between 52 and 144 days. Until the mid-1980s, the total number of bats was usually under one hundred. From the 1990s on, the numbers of *M. myotis* began to increase rapidly, still increasing at the end of the monitoring period (Fig. 1a). The maximum likelihood estimate of the exponential population growth rate of *M. myotis*, corrected for winter severity and duration until the survey, was *g*=9.6*%*, with 95% confidence intervals estimated from the likelihood profile *CI*∈[9.3,9.7] (Fig. 2a). The least-squares estimate of growth rate when not accounting for winter severity or duration was lower than that from the maximum likelihood analysis (*g*=8.5*%*,*CI*∈[7.7,9.3]). Absolute values of residuals from the growth model did not decrease significantly with increasing winter severity (slope of the linear model: *CI*∈[−0.11,0.14], suggesting that the observed variance in bat abundance was not due to local seasonal weather conditions.

A different pattern of population size change was recorded for *R. hipposideros* (Fig. 1b), with the number of *R. hipposideros* remaining low during the first half of the monitoring period (8-37 individuals recorded annually between 1977 and 1998), followed by a continuous upward trend from 1999 onwards. Despite the delayed onset of population increase, the exponential growth rate of *R. hipposideros* was higher than that of *M. myotis* at *g*=13.1*%*, *CI*∈[12.4,13.7] (Figs. 1b, 2b). Growth rate without considering winter severity and duration was statistically identical. The likelihood profile of the slope of the linear model describing absolute values of residuals from the growth model relative to winter severity was less than zero (*CI*∈[−0.13,−0.03], Fig. 2b); hence variance in *R. hipposideros* abundance decreased in more severe winters, as hypothesised.

### Roost selection and clustering behaviour

**Fig. 3:**
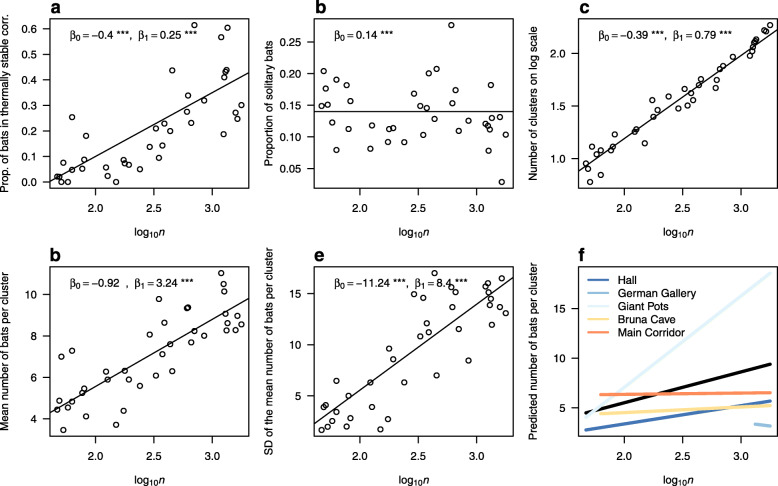
Clustering behaviour and cluster size of hibernating *M. myotis*. Relationship between clustering behaviour (**a**, **b**, **c**) and cluster size (**d**, **e**) characteristics dependent on the number of bats hibernating in the Rock Bull Cave (*n*). The models in panels **a**-**e** correspond to the best linear models explaining the characteristic (Table 1). The coloured lines in panel **f** correspond to the linear models of cluster size relative to the bat abundance. The line extent on the x axis corresponds to the occurrences, when bats utilised the given part of the cave. The black line is the overall null model. *β* = linear model coefficients, *** = *p*<0.001

Each bat species utilised the monitored parts of the cave differently, with warm corridors, notably the Main Corridor (Fig. 1d), providing roosting sites for *R. hipposideros* throughout the survey period (proportion of bats roosting in the warm corridors: 93-100%), while *M. myotis* occurred in both warm and cold sections, with the proportion of bats found in the warm corridors ranging from 0 to 61%. The proportional increase in bats found in the warm corridors is best explained by the increasing abundance of the species (*F*_1,33_=37.96,*p*<0.001; Table 1, Fig. 3a).

**Table 1:** Model selection for characteristics of clustering behaviour in *M. myotis*, where the null model is that of the Eq. 2

Response variable	Final model	AIC_0_	AIC_1_	*Δ*AIC
Proportion of bats in warm corridors	→1+log10*n*	-37.48	-45.83	8.35
Proportion of solitary bats	→1	-102.01	-111.38	9.37
Number of clusters on log_10_ scale	→1+log10*n*	-67.77	-75.09	7.32
Mean number of bats per cluster	→1+log10*n*	118.02	110.67	7.35
Standard deviation of mean cluster size	→1+log10*n*	180.75	173.26	7.49

As *R. hipposideros* do not hibernate in clusters (i.e. where animals touch one another) we only analysed clustering behaviour in *M. myotis*. In this study, most *M. myotis* hibernated in clusters, with the overall proportion of solitarily hibernating individuals being 13.6% (*t*=17.11,*p*<0.001). This value showed no significant change in response to number of hibernating bats, winter severity or duration (Table 1, Fig. 3b).

With the increasing number of bats in the hibernaculum, *M. myotis* predominantly formed more clusters (*F*_1,33_=917,*p*<0.001; Table 1, Fig. 3c). To a less pronounced degree, the average size of clusters was also dependent on the total number of hibernating *M. myotis* in the cave (*F*_1,33_=75.78,*p*<0.001; Table 1, Fig. 3d), with cluster size becoming more variable as the number of hibernating bats increased (*F*_1,33_=85.0,*p*<0.001; Fig. 3e). Modelling the increase in cluster size in response to the increase in bat abundance in different parts of the cave showed that the relationship spatially varies within the hibernaculum (likelihood ratio test: −2*Δ* ln*L*=2123.5,*df*=8,*p*<0.001; Fig. 3f). We found that the increase in cluster size was most pronounced in the Giant Pots corridor (likelihood ratio test: −2*Δ* ln*L*=1440.9,*df*=2,*p*<0.001), where the largest cluster of *M. myotis* was also found (maximum number of individuals = 140).

No significant relationship was observed between either clustering behaviour or cluster size and winter severity or winter duration for *M. myotis*.

## Discussion

Because of the high species richness of hibernating bats found there, the Bull Rock Cave is considered one of the most important bat hibernacula in the Czech Republic and surrounding Central European countries. The species registered include a number rarely found in Central European underground shelters, such as *R. ferrumequinum*, *M. dasycneme*, *P. pipistrellus* and *N. noctula*. As regards bat abundance, the cave is the most significant hibernation site in the country for *M. myotis* (cf. [41]). The trends showing an increase in bat abundance over the monitoring period are congruent with other records from the region. While numbers of hibernating bats declined dramatically in Western and Central Europe over the 1960s and 70s [24, 25, 28, 47–49], an increase in numbers has been recorded since the 1990s, particularly in Central Europe [7, 27, 29, 30, 32, 34, 37, 38, 40, 42]. Population recovery appeared earlier in *M. myotis* than *R. hipposideros*. The sole exception is the recent reversal of the negative trend in *R. hipposideros* abundance noted in Hermann’s Cave in Austria [33]. Changes in numbers of the two dominant bat species recorded in the Bull Rock Cave are similar to those reported elsewhere in Central Europe, though the rate of population growth appears to be exceptionally high (Fig. 1).

Spatial patterns of where bats roost in relation to thermal stability and relative warmth of corridors in the Bull Rock Cave provided insights into species-specific behaviour during hibernation. A stable fraction of hibernating bats showed solitary roosting behaviour in the cave irrespective of any tested predictors. *Myotis myotis* that huddled in torpor showed increased clustering (from four to ten individuals per cluster; Fig. 3d), and began roosting in warm sections of the cave, as their abundance increased (Fig. 3a). Hibernating in small clusters is common in *M. myotis* [18]. There could be three, mutually non-exclusive, explanations derived from research of other bat species for why *M. myotis* form new small clusters in relation to increasing abundance but not to winter severity or duration (Table 1). First, *M. myotis* bats join clusters up to a limit, where the disturbance in the cluster outweights the benefits of huddling. Second, close contact increases pathogen transmission and forming large clusters is selected against in presence of a pathogen. Third, increase in bat population size means that bats survive better and arrive to hibernacula in better conditions, where heavier bats tend to roost solitarily at warmer roosts.

Thermal imaging has shown that the maximum temperature of arousing *M. myotis* bats in a cluster of nine or more individuals increases steeply [50]. When few individuals arouse at the same time, they frequently do so in a cold arousal with increasing their temperature by less than 10 ^∘^C. Increase in body temperature is expensive for bats in torpor, as arousals cost most energy exerted during hibernation [1, 2, 15]. Though clustering reduces heat loss [16] and evaporative water loss [18], hibernating in large clusters could mean that bats risk disturbance from other individuals, resulting in cascading arousals. Rare in *M. myotis*, arousal cascades involve multiple individuals warming up in tandem [21, 50, 51]. Coupled with the observations of higher temperatures in clusters with more arousing individuals, participating in arousal cascades will consume considerable energy resources of bats that would have otherwise remained torpid and could have aroused later in a cold arousal. Higher temperature at arousal and increase in arousal frequency both contribute negatively to the overall energy expenditure balance and influence putative benefits of clustering. Our data do not allow quantification of energy expenditure linked to cluster size, but conspecific disturbance need to be considered in conjunction with the second proposed explanation involving pathogen transmission in clusters.

Arousal cascades occur in Nearctic *Myotis lucifugus* infected with *Pseudogymnoascus destructans* more frequently than in *M. myotis* (33% [21] or 78% [51] of all arousals are arousal cascades in infected *M. lucifugus* vs. 13% in *M. myotis* [50]). The majority of hibernating bats in the Bull Rock Cave were also infected with *P. destructans*, because regional infection prevalence approaches 100% [52]. The fungus *P. destructans* causes white-nose syndrome (WNS), an emerging disease that has affected hibernating bats in the eastern Nearctic since 2006 [53, 54]. Its rapid spread has resulted in mass mortalities and precipitous population declines, resulting in up to 10-fold decreases in the abundance of multiple bat species in hibernacula and causing extensive local extinctions [55–59]. Large agglomerations of hibernating bats in Nearctic hibernacula, particularly in natural caves, are likely to be one of the main factors contributing to the rapid spread of the disease [58]. Contrary to the Nearctic, the disease appears to be endemic in the Palearctic [52, 60], where affected bats have evolved to tolerate the infection [52, 61]. The impact of the disease eliminated large differences in species abundance patterns that existed between the Palearctic and Nearctic hibernacula prior to the disease’s emergence and the Nearctic bats form smaller agglomerations in hibernacula now than they did prior to WNS emergence [58]. *Myotis myotis*, which have been most severely affected by WNS in the Palearctic [62], have been shown to mount an energetically costly systemic response to invasive infection [63]. A preference for colder roosts is in agreement with the hypothesis that roosting at lower temperatures may be an adaptive response to the presence of *P. destructans* as the fungus grows slower in cold conditions [14]. In regions with high prevalence of *P. destructans* infection, *M. myotis* that choose to hibernate predominantly in colder corridors in small clusters will benefit from reduced pathogen pressure.

We observed the largest clusters of *M. myotis* bats in the cold Giant Pots corridor, but the bats did not cluster more with increasing abundance in other parts of the cave (Fig. 3f). In the Giant Pots corridor, the bats frequented the deep hollows formed by water erosion in the ceiling of the corridor in roosts that are similar to sheltered chimneys without draught preferred by the species at other sites [64]. Elsewhere in the Bull Rock Cave, the bats extensively utilised warm parts of the cave with increasing abundance (Fig. 3a), forming new clusters (Fig. 3c). The third proposed explanation for the clustering behaviour of *M. myotis* addresses the discrepancy of why bats form new clusters in warm microclimate instead of joining existing clusters in cold roosts. Heavier bats tend to hibernate solitarily in warm parts of hibernacula [16]. We do not have longitudinal data on bats’ weight at the beginning of hibernation, but let’s assume that at the times of increasing population size, individuals survive better and thus start hibernating with greater fat resources. Progressively greater number of bats hibernated in warm parts of the cave (Fig. 3a), but the proportion of solitary roosting individuals remained statistically stable (Fig. 3b).

In contrast, *R. hipposideros* hibernate predominantly in the warmer corridors and react more to weather conditions. The error in population size prediction in our model decreased in more severe winters, suggesting that the model was better at predicting the number of bats hibernating in the cave when severe winter conditions prevented flight activity. Bats spend more time in the hibernacula and in words of Kerbiriou et al. [35], hibernacula become more attractive for *R. hipposideros* in severe winters. *Rhinolophus hipposideros* is close to its northern distribution limit at the study site [65], and it encounters higher temperatures in hibernacula at the core of its range in Southern Europe [32, 66, 67]. Hibernating farther north in Central Europe, where climate is colder, requires that *R. hipposideros* prefer warm roosts with stable temperature in the Bull Rock Cave.

The pathogen pressure elicited by *P. destructans* on *R. hipposideros* bats is less than that in *M. myotis*, because about 80% of individuals are infected and out of those about half develop skin lesions indicative of WNS [52, 62]. Furthermore, being solitary hibernators, *R. hipposideros* are less affected by bat-to-bat pathogen transmission during hibernation.

## Conclusions

Our results show increase in abundance of hibernating *M. myotis* and *R. hipposideros* and species-specific hibernation preferences. The size of hibernating *M. myotis* clusters is dependent on the total number of bats found in the hibernaculum, though only slight variation in the average cluster size was observed over the monitoring period. With about ten individuals per cluster when almost 1800 bats were present in the hibernaculum, the small relative cluster size is an inherent hibernating strategy of the species, possibly a result of co-evolution with the pathogenic fungus causing WNS and behavioural response to disturbance from neighbours. In hibernating *R. hipposideros*, animals do not huddle, and form agglomerations in warm corridors. Considering changes in abundance, more *R. hipposideros* are found in hibernacula in severe winters.

## Methods

### Study site

The Bull Rock Cave (Býčí skála in Czech) is one of the largest limestone underground cave systems in the Czech Republic, with a total passage length approaching 15 km. The best-known part of the cave is called Old Bull Rock and, with the total length of about 500 m, the Old Bull Rock Cave is famous for archaeological discoveries associated with the Iron Age Halstatt culture [68, 69]. The main entrance to this part of the cave lies at 306 m a. s. l. (49.31 N, 16.69 E). New parts of the cave have been discovered after 1920, when the ceiling of the water sink Šenk was removed, and speleologists continue to penetrate new sections to this day. To date, individual *R. hipposideros* and *M. mystacinus* bats have been sporadically observed hibernating in the newly opened sections following the Šenk sink.

The data examined in this study were all gathered in the 500m long tunnel flow cave. Bats were counted separately in several distinct parts of the cave. The Hall (Entrance Hall, Halstatt Hall, Předsíň in Czech) is a large domed space at the cave entrance, with a single narrow deviating passage. This space is connected with the aboveground environment through a natural window and an artificially opened corridor. The Hall was restructured as an underground arms factory during World War II, but the machinery has since been removed, leaving concrete platforms on the cave floor. The Giant Pots (Obří hrnce in Czech) are an adjacent space, connected to the second artificial entrance with a broadened corridor (The German Gallery). The Giant Pots passage is about 25m long, with a ceiling modelled by water erosion into a number of deep hollows called pots. The jagged Bruna Cave system deviates from the Giant Pots space at the mouth of the Heathen (Pagan) Chimney, forming a network of narrow passages in the higher floor of the cave. The Giant Pots continue further into the Main Corridor, running about 300m up to the Šenk Sink. The Main Corridor is a spacious passage, widening at the openings of large vertical chimneys and diverging into many small side deviations. The Hall, the German Gallery and the Giant Pots sections are thermally unstable across seasons, with temperatures varying between 1 and 5 ^∘^C during the surveys, while the Bruna Cave and the Main Corridor are thermally stable with temperatures varying between 6 and 8 ^∘^C (Fig. 1d; [43, 46]). The two time periods when temperature was measured inside the cave (1980’s and 2000’s) indicate parts of the cave that could be considered cold and warm, respectively. The Hall, the German Gallery and the Giant Pots sections are referred to as cold parts of the cave, and the Bruna Cave and the Main Corridor as warm.

### Bat surveys

Annual bat number surveys have been performed regularly in late February or early March since 1977, though the first (1977) survey was on 22 January. The senior author attended all the monitoring trips, guaranteeing that a standard route was always followed through the sections monitored. The bat survey was conducted visually, using a telescope and a focused flash light. As the bats were not handled for examination of species characteristics, it was not always possible to determine species status. Numbers of each species (where possible) or genus (where species identification was ambiguous) were recorded in each section separately. The number of *M. myotis* bats in each cluster was counted separately throughout the study. Bats were considered clustered when roosting individuals were touching one another.

### Calculation of winter duration and severity

In order to account for annual differences in weather, we calculated winter duration and severity according to the accumulated winter season severity index (AWSSI) [70]. In the AWSSI calculation, the winter season starts on the earliest day when either daily maximum air temperature is ≤0^∘^C, snow depth is ≥0.25cm or the date is 1 December. Winter ends on the last day when either daily maximum air temperature is ≤0^∘^C, daily snowfall is ≥0.25cm, snow depth is ≥2.5cm or the date is 1 March. Between the winter start and end dates, the AWSSI increases incrementally relative to air temperature, snowfall and snow depth. Snowfall is calculated in the AWSSI algorithm from daily temperature extremes and precipitation. We obtained daily summary data on air temperature, precipitation and snow depth from the Tuřany meteorological station through the Global Historical Climatology Network [71, 72]. We imputed missing values for average, minimum and maximum daily temperatures through predictive mean matching. Missing precipitation data was conservatively set to zero, while missing snow depth was estimated within the AWSSI calculation, taking into account snowfall and snow melting [70] (Additional file 2).

### Statistical analysis

To investigate drivers of changes in population size for bat species hibernating in the Bull Rock Cave, we developed a likelihood model that co-estimated population size change parameters within the model, together with model parameters of possible drivers. Following inspection of the data, we modeled population size changes with an exponential curve that predicts continuous growth. Exponential growth was modelled as follows:

1 $$  \hat{n} = ab^{t-c},  $$

where $\hat {n}$ is the predicted number of bats, *t* is time in calendar years, and *a*,*b*,*c* are model parameters with *c*=1977 to translate the model to calendar years. The population growth rate per year (%) is then given as *g*=(*b*−1)×100.

**Influence of winter duration.** We model the influence of winter duration on survey numbers as follows. Increased flight activity will reduce hibernating survey numbers, and flight activity increases with the interval between peak midwinter and survey date. Thus, absolute values of residuals from the population size model are expected to increase with later sampling date.

**Influence of winter severity.** Stochastic departures from the model predictions will be observed in milder winters, and bat counts will be relatively stable in more severe winters as flight activity is reduced in adverse environmental conditions. Absolute values of the population size model residuals are expected to decrease with increasing winter severity.

The likelihood model accounts for the influence of seasonal dynamics and the timing of bat count surveys. The total log-likelihood of the number of bats surveyed in a year accounting for winter severity and winter duration until survey is given as

$$\ln L(n|t,w,d) = \ln L(n|t) + \ln L(r|t,w,d), $$

where *L* is likelihood, *n* is the observed number of bats, *w* is the accumulated winter season severity index on the survey date [70], *d* is duration of winter from its onset to the survey date (see “Calculation of winter duration and severity” section for more details), and *r* is the absolute value of the residual from the population size model (Eq. 1).

The log-likelihood of the observed number of bats is the sum of log-probabilities of bat observations given the population size model prediction under Poisson distribution in each year:

$$\ln L(n|t) = \sum\limits_{t=1977}^{2018}{\ln \mathcal{P} \left(\hat{n}_{t}\right)} = \sum\limits_{t=1977}^{2018}{\ln \frac{\hat{n}_{t}^{n_{t}} e^{-\hat{n}_{t}}}{n_{t}!}}. $$

The log-likelihood of absolute values of residuals from the population size model is the sum of the log-probability of absolute values of residuals given by the winter severity model and the winter duration model under normal distribution:

$$\begin{array}{*{20}l} \ln L(r|t,w,d) =& \sum\limits_{t=1977}^{2018}{\ln \mathcal{N} \left(\hat{r}_{w_{t}}, \left(\hat{r}_{d_{t}}\right)^{2}\right)} \end{array} $$

$$\begin{array}{*{20}l} =& \sum\limits_{t=1977}^{2018}{\ln \frac{1}{\sqrt{2\hat{r}_{w_{t}} \left(\hat{r}_{d_{t}}\right)^{2}}} e^{-\frac{\left(r_{t} - \hat{r}_{w_{t}}\right)^{2}}{2\left(\hat{r}_{d_{t}}\right)^{2}}}}, \end{array} $$

where $\hat {r}_{w}$ is the predicted value of *r* from a linear model dependent on winter severity *w*, and $\hat {r}_{d}$ is the predicted value of *r* from a linear model dependent on winter duration *d*. The parameters *a*_*w*_ and *b*_*w*_ from the linear model relating *r*→*w* were co-estimated with the parameters *a* and *b* of the population size model (Eq. 1). The parameters *a*_*d*_ and *b*_*d*_ from the model *r*→*d* were fixed to those relating residuals from the growth model fitted with the least-squares estimator to reduce dimensionality of the likelihood search.

### Cluster size

In each year, we analysed the proportion of bats roosting in warm corridors, proportion of solitary roosting bats, the number of clusters, the mean number of bats per cluster and the standard deviation of the mean number of bats per cluster. We evaluated relationships within the variables characterising clustering of *M. myotis* with the total number of *M. myotisn* on a log scale, and with winter severity *w* and winter duration *d* using multiple linear models. We ran the models separately for each response variable characterising clustering and selected explanatory variables with backward selection based on the Akaike Information Criterion (AIC). The null model was parametrised with a least-squares estimator as a function mapping:

2 $$  x \!\to\! 1+ \log_{10} n + w + d + \log_{10} n\!:\!w + \log_{10} n:d + w\!:\!d,  $$

where *x* is one of the variables characterising clustering, and the symbol : denotes interaction of the terms.

We evaluated the change in the number of bats in clusters located in different parts of the cave in response to changes in bat abundance using likelihood ratio tests. Having the overall linear relationship as the null model, we calculated the ln*L* of the alternative model as the sum of log-likelihoods of linear models fitted with least-squares on data from parts of the cave (Hall, German Gallery, Giant Pots, Bruna Cave and the Main Corridor). To test whether the relationship between cluster size and bat abundance differs in the Giant Pots corridor, the sum of ln*L* of the linear model from the Giant Pots and ln*L* of all other parts of the cave constituted the alternative model. The designs of both tests are nested, making the likelihood ratio test appropriate.

All analyses were run in the R statistical framework [73] using the mice package [74] for predictive mean matching. The cluster size data and scripts are available in the Additional File 3.

## Supplementary information

**Additional file 1** Number of bats surveyed in the Bull Rock Cave hibernaculum between 1977 and 2018. The table shows the total number of *Myotis myotis* (Mmyo) and *Rhinolophus hipposideros* (Rhip) hibernating in the cave each year. Data on other species are available for a subset of years, including those from [43]. Mdau = *Myotis daubentonii*, Mdas = *Myotis dasycneme*, Mema = *Myotis emarginatus*, Mnat = *Myotis nattereri*, Mmys = *Myotis mystacinus*, Mbech = *Myotis bechsteinii*, Mbra = *Myotis brandtii*, Ppip = *Pipistrellus pipistrellus*, Bbar = *Barbastella barbastellus*, Paur = *Plecotus auritus*, Paus = *Plecotus austriacus*, Nnoc = *Nyctalus noctula*, Eser = *Eptesicus serotinus*, Date = survey date, awssi.winter = Accumulative Winter Season Severity Index [70] on the date surveyed (*w*), sampling.winter = duration of winter up to the survey date (*d*).

**Additional file 2** R implementation of the calculation of the accumulative winter season severity index [70]. The script requires data from the Global Historical Climatology Network [71, 72] downloaded in metric units.

**Additional file 3** R scripts and data for likelihood profiling of population size changes conditional on winter severity and duration, and analyses of cluster size of hibernating bats. uses data in the Additional file 1.

## Data Availability

The datasets supporting the conclusions of this article are included within the article and its additional files.
